# SPRIT: Identifying horizontal gene transfer in rooted phylogenetic trees

**DOI:** 10.1186/1471-2148-10-42

**Published:** 2010-02-13

**Authors:** Tobias Hill, Karl JV Nordström, Mikael Thollesson, Tommy M Säfström, Andreas KE Vernersson, Robert Fredriksson, Helgi B Schiöth

**Affiliations:** 1 Department of Neuroscience, Biomedical Centre, Uppsala University, Box 593, SE-751 24 Uppsala, Sweden; 2 Department of Evolution, Genomics and Systematics, Uppsala University, Norbyvägen 18C, SE-752 36 Uppsala, Sweden

## Abstract

**Background:**

Phylogenetic trees based on sequences from a set of taxa can be incongruent due to horizontal gene transfer (HGT). By identifying the HGT events, we can reconcile the gene trees and derive a taxon tree that adequately represents the species' evolutionary history. One HGT can be represented by a rooted Subtree Prune and Regraft (RSPR) operation and the number of RSPRs separating two trees corresponds to the minimum number of HGT events. Identifying the minimum number of RSPRs separating two trees is NP-hard, but the problem can be reduced to fixed parameter tractable. A number of heuristic and two exact approaches to identifying the minimum number of RSPRs have been proposed. This is the first implementation delivering an exact solution as well as the intermediate trees connecting the input trees.

**Results:**

We present the SPR Identification Tool (SPRIT), a novel algorithm that solves the fixed parameter tractable minimum RSPR problem and its GPL licensed Java implementation. The algorithm can be used in two ways, exhaustive search that guarantees the minimum RSPR distance and a heuristic approach that guarantees finding a solution, but not necessarily the minimum one. We benchmarked SPRIT against other software in two different settings, small to medium sized trees i.e. five to one hundred taxa and large trees i.e. thousands of taxa. In the small to medium tree size setting with random artificial incongruence, SPRIT's heuristic mode outperforms the other software by always delivering a solution with a low overestimation of the RSPR distance. In the large tree setting SPRIT compares well to the alternatives when benchmarked on finding a minimum solution within a reasonable time. SPRIT presents both the minimum RSPR distance and the intermediate trees.

**Conclusions:**

When used in exhaustive search mode, SPRIT identifies the minimum number of RSPRs needed to reconcile two incongruent rooted trees. SPRIT also performs quick approximations of the minimum RSPR distance, which are comparable to, and often better than, purely heuristic solutions. Put together, SPRIT is an excellent tool for identification of HGT events and pinpointing which taxa have been involved in HGT.

## Background

Phylogenetic trees are commonly used in evolutionary biology to represent the evolution of a set of extant species. Trees are an appropriate representation of evolutionary history when dealing with species where genes are strictly vertically inherited. However, there are a rapidly growing number of well-supported cases of horizontal gene transfer [1], and thus a need for the development of tools for detecting and identifying specific HGT events.

Introduced to evolutionary biology by Hein [2] the graph-theoretical operation "rooted subtree prune and regraft" (RSPR) is recognized as a way to understand and represent reticulate evolution [3-6]. Loosely described, an RSPR prunes a subtree of a rooted tree and then reattaches it to another part of the tree.

Given any two incongruent rooted phylogenetic trees where the incongruence can be explained by a single reticulation event, one tree can be constructed from the other by a single RSPR. If more than one reticulation event is needed to explain the incongruence, the events can be modeled by a series of RSPRs. Assuming that the two gene trees are correct, the minimum number of RSPRs between them (i.e. their RSPR tree-to-tree distance) gives a lower bound on the number of reticulation events required to reconcile their topologies. For any two gene trees there may be a number of minimal RSPR solutions.

The general problem of calculating the minimum number of RSPRs is NP-hard, but it is also shown that when parameterized by the distance between the two trees, calculating the RSPR distance is fixed-parameter tractable [7]. Reticulation events are relatively rare in biology, indicating that in many biologically relevant cases the number of RSPRs will be small enough to be found within reasonable time. Two 3-approximation algorithms to the minimum RSPR problem are suggested [8,9], however both of them are actually 5-approximations [10]. A novel 3-approximation algorithm and a fixed parameter tractable exact solution are reported in [11]. Another exact solution and implementation, SPRDist, is reported in [12].

Our two main goals in this paper are to present a novel algorithm based in part on the findings in [7] together with its implementation in software and to provide a benchmark comparisons of both exhaustive and heuristic software estimating the minimum number of RSPRs between a pair of trees, i.e. the RSPR problem. The software, SPRIT (SPR Identification Tool, see additional file 1) determines the minimum number of RSPRs needed to transform one rooted binary phylogenetic tree into another. In this section, we give the formal definitions needed to describe the algorithm as well as some additional background.

The definitions follow those of [7,13].

Let T be a rooted binary phylogenetic *X*-tree. To define the RSPR we append a vertex p at the end of a pendant edge attached to the original root of *T*. Let *u *be a vertex on the path from *p *to *v *and *e = {u, v} *an edge of *T *where e is not incident with *p*. Let *T' *be the rooted binary phylogenetic tree obtained from *T *by deleting *e *and then adjoining a new edge *f *between *v *and the component *C*_*u *_that contains *u *by:

i. creating a new vertex *u' *which subdivides an edge in *C*_*u*_, and adjoining f between *u' *and *v*, and

ii. contracting the degree-two vertex *u*

A single RSPR obtained *T' *from *T*.

The RSPR distance between two rooted phylogenetic *X*-trees *T*_1 _and *T*_2 _is defined to be the minimum number of RSPR needed to transform one tree into the other. This distance is denoted *d*_RSPR _(*T*_1_, *T*_2_).

Let *T *be a rooted binary phylogenetic *X*-tree. The neighbourhood *N *of *T *is defined to be all rooted binary phylogenetic *X*-trees, which can be constructed by performing one RSPR on *T*.

**Figure 1:**
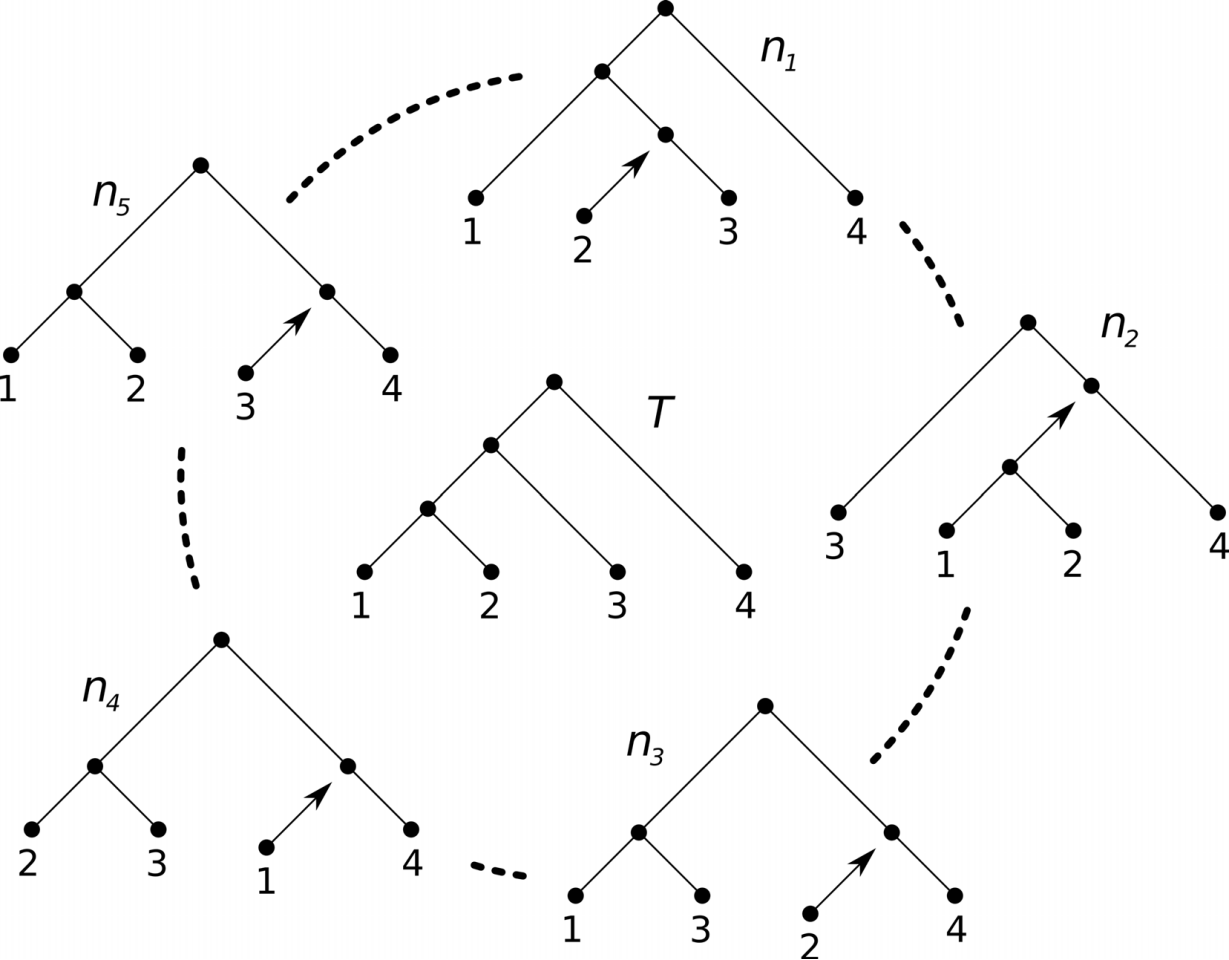
**The figure depicts a set of rooted, binary *X-trees***. The five trees *n*_1-5 _are all separated from *T *by a single RSPR and hence a subset of *T*:s neighborhood *N*. In *n*_2 _and *n*_5_, the branch holding taxa 1 and 2 can be collapsed with respect to *T*.

Let *T *and *T' *be rooted binary phylogenetic *X*-trees. Any pendant subtree *t *that occurs in both *T *and *T' *is replaced by a single leaf *l *with a new label in both *T *and *T' *[7,14]. We denote this process collapsing subtree t on *T *and *T'*. In Figure 1, trees *n*_2 _and *n*_5 _both have a subtree containing taxa 1 and 2 that can be collapsed with regard to tree *T*.

**Figure 2:**
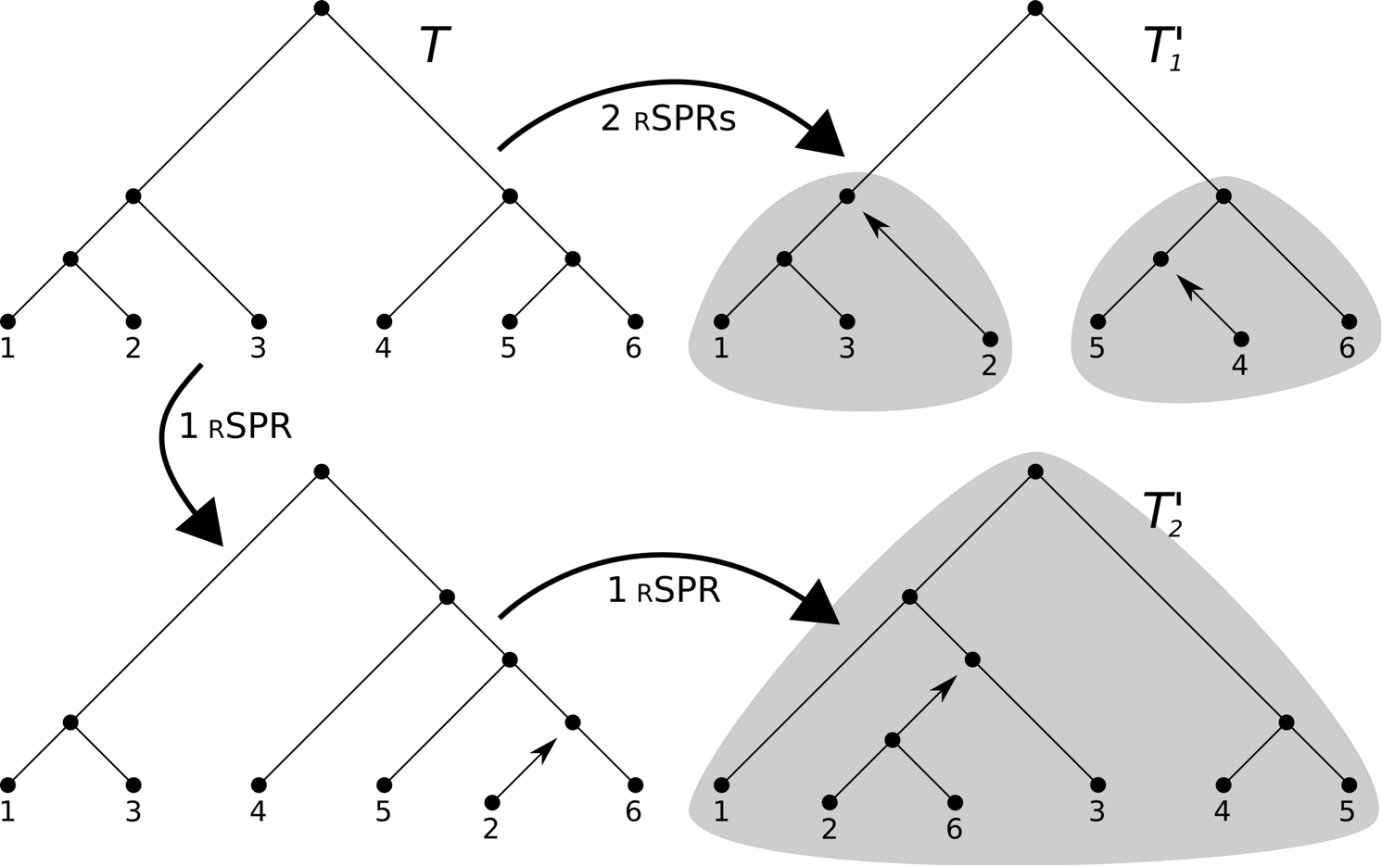
**The two X-trees *T'*_1 _and *T'*_2 _to the right in the figure are both distanced to *T *by two RSPRs**. The shadowed areas mark the sub problems to be solved by the algorithm. *T'*_1 _can be subdivided into two small problems, while *T'*_2 _cannot be subdivided.

Cumulative RSPRs are sets of RSPRs that operate on the same taxa in succession creating entangled RSPRs, i.e. cycles of genetic inheritance. Tree *T'*_2 _in Figure 2 is separated from tree *T *by two cumulative RSPRs, while tree *T'*_1 _has two non-cumulative RSPRs separating it from *T*.

Let *T *and *T' *be rooted binary phylogenetic *X*-trees. Pendant subtrees occurring in *T *and *T'*, including a minimum set of the same taxa, but with non-identical topology, is defined to be a minimal common cluster (*MCC*) of *T *and *T'*.

Closely related to the minimum RSPRs distance is the hybridization number of two trees. The hybridization number is defined to be the minimum RSPR distance when no cumulative RSPRs are included. *MCC *is a sound method to reduce the computational time when calculating the hybridization number, but it does not always preserve the RSPR distance [7]. However, as being able to subdivide the RSPR problem has the potential to reduce the time spend on identifying a solution significantly, we have added the following conjecture and an option to calculate solutions based on it in SPRIT.

We conjecture that:

Let *T *and *T' *be rooted binary phylogenetic *X*-trees. Any *MCC *of *T *and *T' *is considered a solvable common cluster (*SCC*) only if the parent of the *MCC *in *T *and *T' *has the same set of taxa. Solving a *SCC *instead of a *MCC *preserves the RSPR distance.

The *SCC *allows us to gain the speed-up from the cluster reduction, while still calculating the correct solution.

The proposed algorithm for identifying the minimum RSPR distance consists of three major operations:

i. Collapsing identical subtrees to reduce the problem size

ii. Divide and conquer by identifying sub-problems in *SCC*s

iii. Depth first search to solve sub-problemsiv.

## Implementation

**Figure 3:**
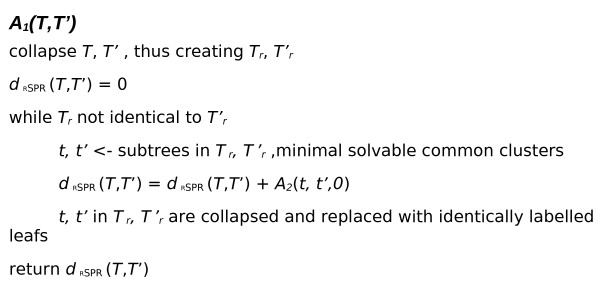
**Pseudo code *A*_1_**. *A*_1 _takes as input two rooted binary phylogenetic *X-trees T *and *T'*. It collapses identical subtrees from *T *and *T' *until only collapsed or non-identical subtrees remain, thus creating the reduced *T*_*r *_and *T'*_*r*_. The minimal solvable common clusters in *Tr *and *T'r *are identified and submitted to *A*2, which calculates the *d*_RSPR _(*t*, *t*') and returns the solution to *A*_1_. The process is repeated until all incongruent subtrees have been submitted to *A*2.

**Figure 4:**
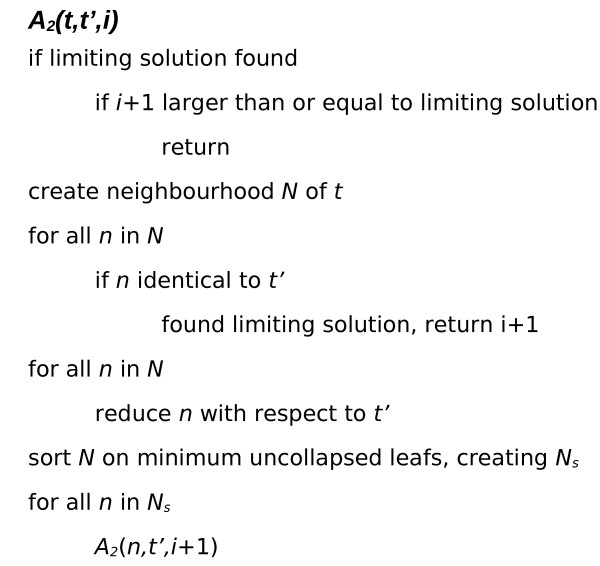
**Pseudo code *A*_2_**. *A*_2 _is recursive and takes as input two incongruent rooted binary phylogenetic *X-trees, t *and *t'*. *A*_2 _performs a greedy but exhaustive, depth first search for *d*_RSPR _(*t*, *t*'). The greedy search quickly identifies an upper limit to the *d*_RSPR _(*t*, *t*'). All possible solutions shorter than the upper limit are evaluated, ensuring a minimal solution.

The algorithm can be broadly divided into two sections, *A*_1 _and *A*_2_. *A*_1 _performs pre-processing, reducing the problem and identifying sub-problems suitable for solving individually. *A*_2 _calculates the RSPR distance between two rooted binary phylogenetic *X-trees T *and *T'*. Pseudo-code describing *A*_1 _and *A*_2 _are given in Figure 3 and 4 respectively. Below is an informal description of *A*_1 _and *A*_2_.

*A*_1 _takes two rooted binary phylogenetic *X-trees T *and *T' *as input (see Figure 3). It proceeds to kernalize the problem by collapsing identical subtrees *t *from *T *and *T' *until only collapsed or non-identical subtrees remain, thus creating the reduced *T*_*r *_and *T'*_*r *_.

Using a divide and conquer approach the solvable common clusters (SCC) with the least number of taxa in *T*_*r *_and *T'*_*r *_are identified. *t *and *t' *are submitted to *A*_2_, which calculates the *d*_RSPR _(*t*, *t*') and returns the solution to *A*_1_.

The subtree *t' *is then collapsed in *T *and the process of collapsing and identifying SCCs is repeated until *T *and *T' *are identical. *A*_1 _returns the sum of the solutions calculated by the calls to *A*_2_.

*A*_2 _is recursive and takes two incongruent rooted binary phylogenetic *X-trees, t *and *t' *as input (see Figure 4). *A*_2 _performs a greedy but exhaustive, depth first search for *d*_RSPR _(*t*, *t*'). The greedy search quickly identifies an upper limit to the *d*_RSPR _(*t*, *t*'). All possible solutions shorter than the upper limit are evaluated, ensuring a minimal solution. The depth first approach reduces memory requirements and the number of cases that need to be evaluated.

In *A*_2_, the neighborhood *N *of *t *is created. Each neighbor *n *in *N *is compared to *t'*, if *n *is identical to *t' *a solution equal to the current level of recursion has been found and is reported as an upper limit. If no identical match to *t' *is found in N, *A*_2 _proceeds to evaluate all neighbors in *N*. Each neighbor in *N *is compared to *t' *and any identical subtrees are collapsed. The number of remaining uncollapsed leaves in each neighbor is used to sort *N *with the lowest number of uncollapsed leaves first, thus creating the sorted neighborhood *NS*. For one *n *in *NS *at a time a recursive call *A*_2 _(*n*, *t'*) is made. This dept first, greedy, recursive search is continued until a limiting solution is found. Once a limiting solution is found an exhaustive search is performed, which will either validate the existing solution or identify new limiting solution. After completing the exhaustive search, *A*_2 _returns the final limiting solution *d*_RSPR _(*t*, *t*').

## Methods and Data

Two data sets were used in this study. The first set was created by randomly performing RSPR on trees as described in [15]. This set was used to extend the study published in [15] and add, since published, exhaustive and heuristic RSPR identification software.

320 pairs of trees were downloaded from the EEEP website [16]. The trees are included in additional file 2. Calculations on the 320 pair data set where limited identically to the original paper, i.e. 4 GB of RAM and 5 hours of running time.

The second data set was produced by manually curating trees to produce non-cumulative RSPRs. A tree containing 5281 taxa was downloaded from the bird supertree project [17]. The tree was manually curated to create a series of 50 trees ranging from 1 to 50 RSPRs distance from the original tree. The curated trees and the original are available in additional file 3. The RSPRs are non-cumulative, i.e. they are not dependent on each other (see Figure 2). The calculations on the bird supertree were limited to 4 GB of RAM and 20 hours of running time.

The following software was included in the benchmark:

EEEP [15] uses evolutionary reasonable constraints on the search space to limit the computations. A strict or permissive ratchet is used to restrict the number of trees investigated further. The trees can also be partitioned into regions of discordance that allows no SPR operations between regions. Rooted, unrooted, bifurcating and multifurcating trees can be processed by EEEP.

HorizStory [18] collapses identical subtrees and performs recursive SPRs until the tree topologies are reconciled. The SPR distance between multifurcating trees can also be calculated using HorizStory.

LatTrans [19] uses a time constraint to ensure that no cycles are introduced when identifying the minimum SPR distance.

PhyloNet's [20] HGT is based on an extended implementation of the RIATA-HGT algorithm [21] Rooted, unrooted, bifurcating and multifurcating trees can be used with PhyloNet.

SPRDist [12] uses integer programming to find the minimum RSPR distance utilizing the connection between the maximum agreement forest (MAF) and the RSPR distance proposed in [8] and later amended by [7].

TNT [22] represents the trees in a matrix of group membership variables with state 1 for members and 0 for non-members. The matrix is used to guide the search for a minimal RSPR path from one tree to the other. Bi- and multifurcating trees are allowed.

HybridInterleave [23] is a Java implementation of the algorithm presented in [24] that calculates the exact number of hybridization events needed to reconcile two binary phylogenetic trees. The minimum number of hybridization events is not identical to the minimum number of RSPR but HybridInterleave was included as it is exact.

Of these eight pieces of software, LatTrans, PhyloNet, EEEP and HorizStory have the option to return multiple solutions, i.e. if there are several RSPR paths with the same distance, several of them will be reported.

All trees used in benchmarking are available in Newick format in additional file 2 and 3.

Any calculation failing due to memory or time constraints was not rerun, but considered a failed attempt. Three different kinds of results were considered in this study. Failed, i.e. the software was either unable to complete the analysis within the given time and memory limitations or crashed during execution. Minimal solution, i.e. the smallest solution found by any software included in the study for a specific tree pair. Solution, i.e. the software reported a solution but not necessarily the minimal solution.

TNT [22] was run at the most sensitive settings of one million iterations and 1000 "stratifications". All other software was run with default settings. On the first test set, SPRIT was run both with five hours and 30 seconds time limit.

## Results

**Figure 5:**
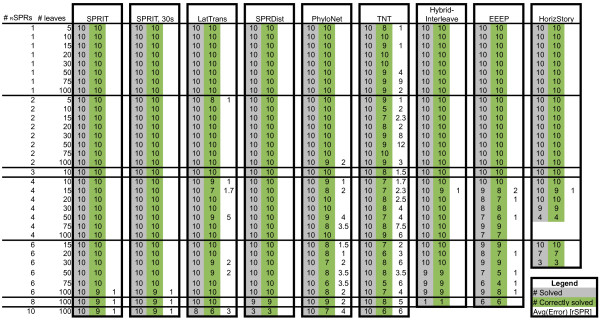
**The table contains the results from the benchmark of SPRIT against LatTrans, SPRDist, PhyloNet, TNT, HybridInterleave, HorizStory and EEEP**. The data for LatTrans, HorizStory and EEEP is adapted from [15], while the data for PhyloNet, TNT, HybridInterleave, SPRDist and SPRIT is new. Each piece of software was given a maximum five hours on each test with the exception of a second run with SPRIT, which was limited to thirty seconds per test. Here, the test set is separated into subsets depending on the number of leaves and the number of separating RSPRs with ten test cases in each subset. The first column gives the number of leaves and the second the number of SPRs between the two trees. The columns for each program give the number of solved trees, the number of correctly solved trees and the average error in RSPRs, in that order.

The benchmark from [15], comparing EEEP, HorizStory [18] and LatTrans [19] was expanded with data for SPRIT, TNT [22], PhyloNet [20], SPRDist [12] and HybridInterleave [23] and the results are presented in Figure 5.

### Small and medium trees

SPRIT delivers solutions to all tree pairs in both 5 h and 30 s setting. For each data set with 100 leaves and six, eight or ten RSPR, SPRIT overestimates the *d*_RSPR _with one RSPR in a single tree pair. PhyloNet and TNT solved all trees but both overestimated *d*_RSPR _with two or more RSPRs in 17 and 47 tree pairs, respectively. LatTrans solved all tree pairs, except two tree pairs with 10 RSPRs, but overestimates *d*_RSPR _with at least two RSPRs in seven cases. SPRDist returns minimal solutions, but fails to deliver solutions for one tree pair with eight RSPRs and seven tree pairs with ten RSPRs. HybridInterleave was only able to complete one tree pair with eight RSPRs and none of the tree pairs with ten RSPRs. EEEP was at best able to solve tree pairs with eight RSPRs and HorizStory at best with six. EEEP successfully returned the minimal solution for all tests with three or less RSPRs or four RSPRs and ten leaves. Among the other tests, seven tree pairs were overestimated with one RSPR and one test with two RSPRs. HybridInterleave and HorizStory both failed to deliver solutions for a number of tree pairs but only overestimated the *d*_RSPR _with one RSPR for a single tree, respectively, when the successful calculations were considered.

LatTrans, PhyloNet, EEEP and HorizStory all have options to identify multiple possible solutions. Considering the subset of 214 tests were these four programs returned the minimal solution, LatTrans and PhyloNet returned the highest number of solutions in 66 cases, which all were shared with the other programs. HorizStory returned the highest numbers of solutions in 191 cases of which 113 were unique to HorizStory. The corresponding numbers for EEEP were 101 solutions with 23 unique to EEEP. The number of solutions returned by LatTrans, PhyloNet and EEEP are generally in the same magnitude, while HorizStory, especially for tests with more than four RSPRs, returns much higher numbers (see additional file 4).

The test was timed and LatTrans, SPRDist, PhyloNet, TNT and EEEP never came close to the five-hour time limit (see additional file 5). Of these five programs, TNT was closest, with a maximum run time of three hours. In addition, when these programs fail, they do so within five hours. SPRIT was time limited to five hours and was forced to use the full time span on the larger trees with six or more RSPRs, but still returned a solution. On the other hand, SPRIT returned the same solution when the time limit was changed from five hours to 30 seconds (see Figure 5). HybridInterleave and HorizStory were the only software's that exceeded the time limit without returning a solution in tests with four, six, eight or ten RSPRs and 30 or more leaves. PhyloNet was the fastest software, with a maximum computing time of 7 seconds.

### Large trees

In addition, SPRIT, PhyloNet, TNT, SPRDist, HybridInterleave and LatTrans were timed performing a search for the minimal RSPRs distance on a very large tree, containing 5281 taxa, manually curated with between 1 and 50 RSPRs (see Table 1). SPRIT was run in exhaustive mode and the times were ranging from three to 15 seconds as the number of RSPRs was raised from one to 50. HybridInterleave solved all test sets in 5 seconds. TNT was also able to calculate all the test sets, but took three minutes with 150 iterations and 100 "stratifications". LatTrans consumed too much memory and was only successful when the number of RSPRs was seven or below, with times between 14 seconds and 65 minutes. SPRDist failed when *d*_RSPR _was above six RSPRs. PhyloNet consumed more than 20 hours per test and was considered unsuccessful.

**Table 1:** A very large tree (5281 taxa) was manually curated to create 50 trees with 1-50 RSPRs distance from the original tree.

#RSPRs	SPRIT	LatTrans	SPRDist	PhyloNet	TNT	Hybrid-Interleave
**1**	3 s	14 s	2 min	-	3 min	5 s
**5**	5 s	7 min	3 min	-	3 min	5 s
**10**	7 s	-	-	-	3 min	5 s
**20**	10 s	-	-	-	3 min	5 s
**30**	12 s	-	-	-	3 min	5 s
**40**	13 s	-	-	-	3 min	5 s
**50**	15 s	-	-	-	3 min	5 s

The table shows the time needed to find the minimum RSPR distance using SPRIT, LatTrans, SPRDist, PhyloNet, TNT and HybridInterleave. Where no data is presented, the computation was aborted due to exceeding 20 h of computation time or 4 GB of RAM.

## Discussion

In this paper, we present SPRIT, a novel algorithm and software implementation that solves the rooted binary *d*_RSPR _minimization problem. SPRIT can be used to identify the exact *d*_RSPR _solution as well as quick approximate solutions. We have compared SPRIT to other software, with heuristic or exact approaches to identifying solutions of the *d*_RSPR_, to evaluate their performance and ability to find correct solutions.

We downloaded a set of 320 tree pairs from [16] and compared the performance of SPRIT to the published performance of EEEP, LatTrans and HorizStory [15,18,19]. We also included PhyloNet [20], TNT [22], SPRDist [12] and HybridInterleave [23] in the benchmark.

The benchmark was included to compare the ability to infer a solution within a specified time and whether the solution found was the minimum solution. SPRIT, PhyloNet and TNT were able to deliver solutions to all tree pairs within the stated limitations. PhyloNet and TNT however have a large margin of error on the delivered solutions compared to SPRIT. LatTrans only fails to solve two tree pairs, but overestimates 11 tree pairs with in total 23 RSPRs. HorizStory, HybridInterleave and SPRDist have none or a single error on the reported solutions, respectively. They are however unable to deliver solutions for 87, 19 and 8 tree pairs respectively. As HorizStory exceeds the five-hour time limit on a number of tree pairs it should be considered that calculating multiple solutions might be more time consuming than returning a single solution. On the other hand, the other three programs calculating multiple solutions do so well within the time limit. Given that HybridInterleave calculates the hybridization number and not the RSPRs distance, we can conclude that these two measures are comparable in most cases.

EEEP, LatTrans, HorizStory and PhyloNet all have options to report multiple solutions if there are more than one minimal RSPR path. As shown in additional file 4, HorizStory returns considerably more solutions for the tests with four or more RSPRs. This is partly because HorizStory permutates the order of RSPRs that effect distinct taxa and returns them as separate solutions. Having a set of equally parsimonious minimal solutions could be beneficial when investigating the course of the reticulation events.

The test sets can be broadly divided into two categories, easy and hard to solve. The easy to solve tree pairs are characterized by lending themselves to a high degree of cluster reduction, i.e. the subtrees where the RSPRs are located are small and the search space therefore limited. The RSPRs in an easy to solve tree pair are generally non-cumulative.

The hard to solve tree pairs have the opposite characteristics i.e. the subtrees where the RSPRs are located are large and the RSPRs are cumulative. This increases the search space and reduces the use of cluster reduction.

Given the rare nature of reticulation events, cumulative RSPRs could be expected to be unusual when dealing with biological data. At the same time, the increasing speeds of computers combined with the continuous growth of available data makes it possible to infer larger phylogenetic trees with higher resolution than before. Here, we represent those circumstances with a large tree containing 5281 taxa manually curated to create a series of 50 trees with 1-50 RSPRs of the simple type (see Figure 2). SPRIT's, LatTrans', SPRDist's, PhyloNet's, TNT's and HybridInterleave's ability to kernalize and solve this RSPRs minimization problem were tested. As shown in Table 1, SPRIT, TNT and HybridInterleave were the only software that could complete the full test set within 20 hours/case using less than 4 GB of RAM. Comparing these results to those of the smaller test set, makes it clear that LatTrans, SPRDist and PhyloNet are limited by the size of the trees as well as the number of RSPRs. SPRIT, TNT and HybridInterleave on the other hand are not limited by the size of the trees but by the number and nature of the RSPRs.

The fixed parameter tractable nature of the *d*_RSPR _minimization problem [7] and the low frequency of reticulation events indicate that SPRIT can be used to quickly and accurately identify the minimum number of RSPRs in very large phylogenies.

## Conclusion

SPRIT's combined ability to always deliver a solution, deal with very large phylogenies, run in heuristic as well as exhaustive mode and a very low rate of overestimation in heuristic mode makes it suitable for identifying RSPRs and involved taxa.

## Availability and requirements

• Project name: SPRIT (SPR Identification Tool)

• Project home page: http://code.google.com/p/phylogenetics/

• Operating system(s): Platform independent

• Programming language: Java

• Other requirements: Java 5 or higher

• License: GNU GPL

• Any restrictions to use by non-academics: none

## Abbreviations

RSPR: Rooted subtrees prune and regraft; *d*_RSPR_: minimum RSPR distance; SPRIT: SPR Identification Tool; EEEP: Efficient Evaluation of Edit Paths; HGT: Horizontal Gene Transfer; NP: Nondeterministic Polynomial; MCC: Minimal Common Cluster; SCC: Solvable Common Cluster.

## Authors' contributions

TH, TS and AV performed initial studies. TH, MT, RF and HS conceived the study and participated in its design. TH and KN participated in its design and in the writing of the manuscript. All authors read and approved the final manuscript.

## Supplementary Material

Additional file 1 **The software, documentation and example files are included in the file.** The most recent version is available at http://code.google.com/p/phylogenetics/. Click here for file

Additional file 2 **320 pairs of trees were downloaded from the EEEP website**[**16**], **they are included in this file.** Click here for file

Additional file 3 **A tree containing 5281 taxa was downloaded from the bird supertree project **[**17**]. The tree was manually curated to produce fifty trees ranging from one to fifty RSPR in distance from the original tree. The file contains the fifty trees and the original. Click here for file

Additional file 4 **LatTrans, PhyloNet, EEEP and HorizStory all return multiple solutions. **Here, the distributions of the number of trees are presented as median [min; max]. The tests have been separated in groups of ten depending on the number of RSPRs and the number of trees. The first column for each program gives the distribution for the correctly solved trees and the second column represents the incorrect solutions. Click here for file

Additional file 5 **The software was timed on each test in the small to medium size test set. **The distributions of calculation time are presented on the form median [min, max]. The first column for each piece of software gives the calculation times for correctly solved tests, the second gives incorrectly solved tests and the third gives the elapsed time when the calculations failed. Click here for file

## References

[B1] Keeling PJ Palmer JD Horizontal gene transfer in eukaryotic evolution Nature reviews 2008 9 8 605 618 10.1038/nrg2386 18591983

[B2] Hein J Reconstructing evolution of sequences subject to recombination using parsimony Mathematical biosciences 1990 98 2 185 200 10.1016/0025-5564(90)90123-G 2134501

[B3] Baroni M Grunewald S Moulton V Semple C Bounding the number of hybridisation events for a consistent evolutionary history Journal of mathematical biology 2005 51 2 171 182 10.1007/s00285-005-0315-9 15868201

[B4] Maddison WP Gene trees in species trees Systematic biology 1997 46 3 523 536

[B5] Nakhleh L Warnow T Linder CR St John K Reconstructing reticulate evolution in species-theory and practice J Comput Biol 2005 12 6 796 811 10.1089/cmb.2005.12.796 16108717

[B6] Song YS Hein J Parsimonious Reconstruction of Sequence Evolution and Haplotype Blocks Algorithms in Bioinformatics. vol. Volume 2812/2003 2003 Heidelberg: Springer Berlin 287 302

[B7] Bordewich M Semple C On the computational complexity of the rooted subtree prune and regraft distance Annals of combinatorics 2005 8 4 409 423 10.1007/s00026-004-0229-z

[B8] Hein J Jiang T Wang L Zhang K On the complexity of comparing evolutionary trees Discrete Applied Mathematics 1996 71 1-3 153 169 10.1016/S0166-218X(96)00062-5

[B9] Rodrigues ME Sagot M-F Wakabayashi Y Some Approximation Results for the Maximum Agreement Forest Problem Approximation, Randomization, and Combinatorial Optimization: Algorithms and Techniques. vol. Volume 2129/-1/2001 2001 Heidelberg: Springer Berlin 159 169 full_text

[B10] Bonet ML St John K Mahindru R Amenta N Approximating subtree distances between phylogenies J Comput Biol 2006 13 8 1419 1434 10.1089/cmb.2006.13.1419 17061919

[B11] Bordewich M McCartin C Semple C A 3-approximation algorithm for the subtree distance between phylogenies 2008 6 Elsevier 458 471

[B12] Wu Y A practical method for exact computation of subtree prune and regraft distance Bioinformatics (Oxford, England) 2009 25 2 190 196 10.1093/bioinformatics/btn606 19019848

[B13] Bordewich M Semple C Computing the hybridization number of two phylogenetic trees is fixed-parameter tractable IEEE/ACM transactions on computational biology and bioinformatics/IEEE, ACM 2007 4 3 458 466 10.1109/tcbb.2007.1019 17666765

[B14] Allen BL Steel M Subtree Transfer Operations and Their Induced Metrics on Evolutionary Trees Annals of Combinatorics 2001 5 1 1 15 10.1007/s00026-001-8006-8

[B15] Beiko RG Hamilton N Phylogenetic identification of lateral genetic transfer events BMC evolutionary biology 2006 6 15 10.1186/1471-2148-6-15 16472400 PMC1431587

[B16] EEEP: Efficient Evaluation of Edit Paths http://bioinformatics.org.au/eeep/

[B17] Bird supertree project http://linnaeus.zoology.gla.ac.uk/~rpage/birdsupertree/supertrees/46cb61736e483.tree

[B18] MacLeod D Charlebois RL Doolittle F Bapteste E Deduction of probable events of lateral gene transfer through comparison of phylogenetic trees by recursive consolidation and rearrangement BMC evolutionary biology 2005 5 1 27 10.1186/1471-2148-5-27 15819979 PMC1087482

[B19] Hallett MT Lagergren J Efficient Algorithms for Lateral Gene Transfer Problems RECOMB 2001 2001 Montreal: ACM 149 156 full_text

[B20] Than C Ruths D Nakhleh L PhyloNet: a software package for analyzing and reconstructing reticulate evolutionary relationships BMC bioinformatics 2008 9 322 10.1186/1471-2105-9-322 18662388 PMC2533029

[B21] Nakhleh L Ruths D Wang L RIATA-HGT: a fast and accurate heuristic for reconstructing horizontal gene transfer 2005 3595 Springer 84

[B22] Goloboff PA Calculating SPR distances between trees Cladistics 2007 24 4 591 597 10.1111/j.1096-0031.2007.00189.x 34879631

[B23] Collins L Linz S Semple C Quantifying hybridization in realistic time http://www.math.canterbury.ac.nz/~c.semple/software.shtml 10.1089/cmb.2009.0166 21210735

[B24] Bordewich M Linz S John KS Semple C A reduction algorithm for computing the hybridization number of two trees 2007 3 86 98 19461978 PMC2684132

