# Supplementary material for: SPRIT: Identifying horizontal gene transfer in rooted phylogenetic trees
Source: BMC Evol Biol. 2010 Feb 13;10:42. doi: 10.1186/1471-2148-10-42 (PMC2829038; doi:10.1186/1471-2148-10-42)
Supplement: Additional file 4 — LatTrans, PhyloNet, EEEP and HorizStory all return multiple solutions. Here, the distributions of the number of trees are presented as median [min; max]. The tests have been separated in groups of ten depending on the number of RSPRs and the number of trees. The first column for each program gives the distribution for the correctly solved trees and the second column represents the incorrect solutions. [file 1471-2148-10-42-S4.PDF]

# rSPRs	# leaves	LatTrans		PhyloNet		EEEP		HorizStory	
1	5	1 [1; 2]		1 [1; 3]		1 [1; 5]		1 [1; 4]	
1	10	1 [1; 2]		1 [1; 3]		1 [1; 5]		1 [1; 4]	
1	15	1 [1; 2]		1 [1; 3]		1 [1; 5]		1 [1; 4]	
1	20	1 [1; 2]		1 [1; 3]		1 [1; 2]		1 [1; 4]	
1	30	1 [1; 2]		1 [1; 3]		1 [1; 2]		1 [1; 4]	
1	50	1 [1; 1]		1 [1; 1]		1 [1; 1]		1 [1; 2]	
1	75	1 [1; 2]		1 [1; 3]		1 [1; 2]		1 [1; 4]	
1	100	1 [1; 1]		1 [1; 1]		1 [1; 1]		1 [1; 2]	
2	5	2.5 [1; 4]	4 [4; 4]	3 [1; 9]		1.5 [1; 5]		6 [1; 22]	
2	10	1 [1; 2]		1 [1; 3]		2 [1; 8]		2.5 [2; 8]	
2	15	1 [1; 2]		1 [1; 3]		2 [1; 10]		3.5 [1; 8]	
2	20	1 [1; 2]		1 [1; 1]		2 [2; 4]		2 [1; 4]	
2	30	1 [1; 1]		1 [1; 1]		1 [1; 2]		2 [2; 4]	
2	50	1 [1; 2]		1 [1; 1]		1 [1; 4]		2 [2; 4]	
2	75	1 [1; 1]		1 [1; 1]		1 [1; 1]		2 [2; 4]	
2	100	1 [1; 1]		1 [1; 1]	4 [4; 4]	1 [1; 1]		2 [1; 4]	
3	10	2 [1; 8]		1.5 [1; 12]		5.5 [1; 50]		16 [1; 100]	
4	10	2 [1; 4]	8 [8; 8]	2 [1; 3]	3 [3; 3]	29 [1; 93]		25 [9; 88]	
4	15	1 [1; 6]	6 [6; 12]	1 [1; 2]	5.5 [2; 9]	4.5 [1; 20]	6 [6; 6]	53.5 [37; 154]	26 [26; 26]
4	20	1 [1; 8]		1 [1; 2]		3 [1; 26]	1 [1; 1]	52.5 [6; 130]	
4	30	1.5 [1; 2]		1 [1; 3]		1.5 [1; 4]		24 [12; 94]	
4	50	1 [1; 2]	32 [32; 32]	1 [1; 3]	1 [1; 1]	1.5 [1; 2]	1 [1; 1]	18 [3; 46]	
4	75	1 [1; 1]		1 [1; 1]	1 [1; 1]	1 [1; 1]			
4	100	1 [1; 1]		1 [1; 1]		1 [1; 1]			
6	15	4 [1; 18]		1.5 [1; 18]	4 [3; 5]	13 [3; 71]		231 [11; 7224]	
6	20	2 [1; 6]		1.5 [1; 9]	2 [1; 3]	2 [1; 12]	3 [3; 3]	321 [28; 1939]	
6	30	2 [1; 3]	30 [30; 30]	1 [1; 3]	1 [1; 4]	1 [1; 4]		266 [93; 408]	
6	50	1 [1; 1]	4 [4; 4]	1 [1; 1]	1 [1; 1]	1 [1; 1]	1 [1; 1]		
6	75	2 [1; 4]		1 [1; 3]	2 [1; 3]	1.5 [1; 2]	1 [1; 1]		
6	100	1 [1; 2]		1 [1; 3]	1 [1; 1]	1 [1; 2]	1 [1; 1]		
8	100	1 [1; 16]		1 [1; 3]	1 [1; 1]	1 [1; 2]			
10	100	2 [1; 3]	10 [4; 16]	1 [1; 2]	1 [1; 1]				

Legend

Correct solutions

Incorrect solutions
