# Supplementary material for: SPRIT: Identifying horizontal gene transfer in rooted phylogenetic trees
Source: BMC Evol Biol. 2010 Feb 13;10:42. doi: 10.1186/1471-2148-10-42 (PMC2829038; doi:10.1186/1471-2148-10-42)
Supplement: Additional file 5 — The software was timed on each test in the small to medium size test set. The distributions of calculation time are presented on the form median [min, max]. The first column for each piece of software gives the calculation times for correctly solved tests, the second gives incorrectly solved tests and the third gives the elapsed time when the calculations failed. [file 1471-2148-10-42-S5.PDF]

# rSPRs	# leaves	SPRIT (Correctly solved)	SPRIT (Incorrectly solved)	SPRIT (Failed)	LatTrans (Correctly solved)	LatTrans (Incorrectly solved)	LatTrans (Failed)
1	5	<1s [<1s; <1s]			<1s [<1s; 1s]		
1	10	<1s [<1s; <1s]			<1s [<1s; 1s]		
1	15	<1s [<1s; <1s]			1s [<1s; 2s]		
1	20	<1s [<1s; <1s]			1s [<1s; 2s]		
1	30	<1s [<1s; <1s]			1s [<1s; 2s]		
1	50	<1s [<1s; <1s]			2s [1s; 2s]		
1	75	<1s [<1s; 1s]			1s [1s; 2s]		
1	100	<1s [<1s; 1s]			1s [1s; 2s]		
2	5	<1s [<1s; <1s]			<1s [<1s; 2s]	1s [1s; 1s]	
2	10	<1s [<1s; <1s]			2s [<1s; 2s]		
2	15	<1s [<1s; <1s]			2s [1s; 2s]		
2	20	<1s [<1s; 1s]			1s [1s; 2s]		
2	30	<1s [<1s; 6s]			2s [1s; 2s]		
2	50	11s [<1s; 23s]			2s [1s; 3s]		
2	75	5s [<1s; 1m]			1s [1s; 3s]		
2	100	12s [<1s; 2m]			1s [1s; 3s]		
3	10	<1s [<1s; <1s]			2s [<1s; 2s]		
4	10	<1s [<1s; 1s]			2s [1s; 3s]	4s [4s; 4s]	
4	15	3s [<1s; 21s]			3s [1s; 3s]	3s [2s; 4s]	
4	20	7s [1s; 29s]			2s [1s; 3s]		
4	30	14s [3s; 2m]			2s [2s; 3s]		
4	50	1m [4s; 28m]			2s [2s; 4s]	14s [14s; 14s]	
4	75	7m [33s; 1h]			2s [2s; 4s]		
4	100	53m [3m; 5h]			2s [2s; 5s]		
6	15	3s [<1s; 14s]			2s [2s; 4s]		
6	20	35s [7s; 9m]			3s [2s; 5s]		
6	30	10m [4s; 4h]			4s [3s; 5s]	11s [11s; 11s]	
6	50	5h [17m; 5h]			4s [2s; 6s]	11s [11s; 11s]	
6	75	5h [5h; 5h]			5s [4s; 7s]		
6	100	5h [40s; 5h]	5h [5h; 5h]		7s [6s; 8s]		
8	100	5h [5h; 5h]	5h [5h; 5h]		18s [10s; 20s]		
10	100	5h [5h; 5h]	5h [5h; 5h]		1m [36s; 1m]	10m [9m; 11m]	11m [1s; 22m]

# rSPRs	# leaves	SPRDist (Correctly solved)	SPRDist (Incorrectly solved)	SPRDist (Failed)	PhyloNet (Correctly solved)	PhyloNet (Incorrectly solved)	PhyloNet (Failed)
1	5	<1s [<1s; <1s]			<1s [<1s; <1s]		
1	10	<1s [<1s; <1s]			<1s [<1s; <1s]		
1	15	<1s [<1s; <1s]			<1s [<1s; <1s]		
1	20	<1s [<1s; <1s]			<1s [<1s; 1s]		
1	30	<1s [<1s; <1s]			<1s [<1s; 1s]		
1	50	<1s [<1s; <1s]			1s [<1s; 2s]		
1	75	<1s [<1s; <1s]			2s [1s; 2s]		
1	100	<1s [<1s; <1s]			2s [2s; 5s]		
2	5	<1s [<1s; <1s]			<1s [<1s; <1s]		
2	10	<1s [<1s; <1s]			<1s [<1s; <1s]		
2	15	<1s [<1s; <1s]			<1s [<1s; <1s]		
2	20	<1s [<1s; <1s]			<1s [<1s; 2s]		
2	30	<1s [<1s; <1s]			<1s [<1s; 1s]		
2	50	<1s [<1s; 1s]			1s [1s; 2s]		
2	75	<1s [<1s; 3s]			2s [1s; 2s]		
2	100	<1s [<1s; 4s]			2s [2s; 5s]	3s [3s; 3s]	
3	10	<1s [<1s; 3s]			<1s [<1s; <1s]		
4	10	1s [<1s; 7s]			<1s [<1s; 3s]	<1s [<1s; <1s]	
4	15	<1s [<1s; 9s]			<1s [<1s; 1s]	1s [<1s; 1s]	
4	20	<1s [<1s; 50s]			1s [<1s; 3s]		
4	30	<1s [<1s; 17s]			1s [<1s; 2s]		
4	50	2s [<1s; 8s]			2s [1s; 3s]	4s [4s; 4s]	
4	75	3s [2s; 10s]			3s [2s; 3s]	4s [4s; 5s]	
4	100	7s [2s; 25s]			3s [2s; 4s]		
6	15	6s [<1s; 13s]			<1s [<1s; 1s]	<1s [<1s; <1s]	
6	20	18s [4s; 32s]			1s [1s; 2s]	2s [1s; 2s]	
6	30	9s [1s; 2m]			2s [1s; 2s]	2s [2s; 3s]	
6	50	6s [2s; 8m]			3s [2s; 4s]	3s [2s; 4s]	
6	75	17s [4s; 10m]			5s [3s; 7s]	3s [3s; 4s]	
6	100	22s [8s; 26s]			4s [2s; 5s]	4s [4s; 5s]	
8	100	51s [16s; 14m]		3s [3s; 3s]	5s [3s; 5s]	3s [3s; 3s]	
10	100	32s [22s; 19m]		2s [1s; 4s]	4s [4s; 7s]	5s [5s; 6s]	

# rSPRs	# leaves	TNT (Correctly solved)	TNT (Incorrectly solved)	TNT (Failed)	HybridInterleave (Correctly solved)	HybridInterleave (Incorrectly solved)	HybridInterleave (Failed)
1	5	<1s [<1s; <1s]	43s [43s; 44s]		<1s [<1s; <1s]		
1	10	<1s [<1s; <1s]			<1s [<1s; <1s]		
1	15	<1s [<1s; <1s]	2m [2m; 2m]		<1s [<1s; <1s]		
1	20	<1s [<1s; <1s]			<1s [<1s; <1s]		
1	30	<1s [<1s; <1s]			<1s [<1s; <1s]		
1	50	<1s [<1s; <1s]	6m [6m; 6m]		<1s [<1s; <1s]		
1	75	<1s [<1s; <1s]	13m [13m; 13m]		<1s [<1s; <1s]		
1	100	<1s [<1s; <1s]	15m [15m; 15m]		<1s [<1s; <1s]		
2	5	<1s [<1s; 45s]	44s [44s; 44s]		<1s [<1s; <1s]		
2	10	<1s [<1s; 2m]	2m [2m; 3m]		<1s [<1s; <1s]		
2	15	2m [<1s; 3m]	4m [3m; 4m]		<1s [<1s; <1s]		
2	20	<1s [<1s; <1s]	4m [4m; 5m]		<1s [<1s; <1s]		
2	30	<1s [<1s; 5m]	7m [7m; 7m]		<1s [<1s; <1s]		
2	50	<1s [<1s; 15m]	16m [16m; 16m]		<1s [<1s; <1s]		
2	75	15m [<1s; 26m]			<1s [<1s; <1s]		
2	100	<1s [<1s; 31m]	37m [37m; 37m]		<1s [<1s; <1s]		
3	10	2m [<1s; 3m]	1m [1m; 2m]		<1s [<1s; <1s]		
4	10	2m [<1s; 3m]	3m [2m; 3m]		<1s [<1s; <1s]		
4	15	4m [3m; 5m]	5m [4m; 5m]		<1s [<1s; <1s]	<1s [<1s; <1s]	
4	20	7m [<1s; 10m]	8m [8m; 8m]		<1s [<1s; 2s]		
4	30	11m [6m; 13m]	12m [11m; 13m]		1s [<1s; 2m]		
4	50	20m [1s; 32m]	22m [15m; 28m]		1s [<1s; 22s]		
4	75	38m [<1s; 50m]	38m [36m; 40m]		12s [<1s; 55s]		
4	100	47m [<1s; 1h]	52m [52m; 52m]		6s [<1s; 4m]		
6	15	5m [3m; 6m]	5m [3m; 5m]		<1s [<1s; <1s]		
6	20	8m [7m; 9m]	9m [7m; 12m]		1s [<1s; 38s]		
6	30	12m [<1s; 15m]	17m [15m; 19m]		19s [<1s; 9m]		
6	50	28m [16m; 34m]	39m [38m; 40m]		34m [9s; 2h]		>5h [>5h; >5h]
6	75	58m [15s; 1h]	1h [55m; 1h]		1h [38s; 4h]		5h [5h; 5h]
6	100	1h [1h; 2h]	1h [1h; 2h]		2h [<1s; 5h]		>5h [>5h; >5h]
8	100	2h [58m; 2h]	2h [2h; 2h]		2m [2m; 2m]		>5h [5h; >5h]
10	100	2h [2h; 2h]	2h [2h; 3h]				>5h [5h; >5h]

# rSPRs	# leaves	EEEP (Correctly solved)	EEEP (Incorrectly solved)	EEEP (Failed)	HorizStory (Correctly solved)	HorizStory (Incorrectly solved)	HorizStory (Failed)
1	5	<1s [<1s; <1s]			<1s [<1s; <1s]		
1	10	<1s [<1s; <1s]			<1s [<1s; <1s]		
1	15	<1s [<1s; <1s]			<1s [<1s; <1s]		
1	20	<1s [<1s; <1s]			<1s [<1s; <1s]		
1	30	<1s [<1s; <1s]			<1s [<1s; <1s]		
1	50	<1s [<1s; <1s]			<1s [<1s; <1s]		
1	75	<1s [<1s; 1s]			<1s [<1s; 5s]		
1	100	<1s [<1s; 2s]			<1s [<1s; 2s]		
2	5	<1s [<1s; <1s]			<1s [<1s; <1s]		
2	10	<1s [<1s; <1s]			<1s [<1s; <1s]		
2	15	<1s [<1s; <1s]			<1s [<1s; 3s]		
2	20	<1s [<1s; 13s]			1s [<1s; 6s]		
2	30	<1s [<1s; 24s]			3s [<1s; 8s]		
2	50	<1s [<1s; 3s]			10s [1s; 3m]		
2	75	<1s [<1s; 1s]			21s [1s; 3m]		
2	100	2s [<1s; 5m]			10s [4s; 16m]		
3	10	<1s [<1s; <1s]			<1s [<1s; 6s]		
4	10	<1s [<1s; 28s]			1s [<1s; 15s]		
4	15	<1s [<1s; 2m]	3s [3s; 3s]	<1s [<1s; <1s]	14m [13m; 16m]	7m [7m; 7m]	
4	20	<1s [<1s; 2m]	<1s [<1s; <1s]	1s [1s; 1s]	3m [4s; 3h]		
4	30	<1s [<1s; 17s]		<1s [<1s; 1s]	12m [2m; 3h]		N/A [N/A; N/A]
4	50	1s [<1s; 28s]	4s [4s; 4s]	2s [2s; 21s]	46m [4m; 2h]		N/A [N/A; N/A]
4	75	8s [2s; 25s]		16s [16s; 16s]			N/A [N/A; N/A]
4	100	15s [4s; 49s]		10s [3s; 13s]			N/A [N/A; N/A]
6	15	<1s [<1s; 2s]		<1s [<1s; <1s]	3m [4s; 10m]		
6	20	2s [<1s; 12s]	4s [4s; 4s]	3s [<1s; 5s]	54m [3m; 5h]		N/A [N/A; N/A]
6	30	3s [<1s; 33s]		16s [16s; 16s]	4h [1h; 5h]		N/A [N/A; N/A]
6	50	7s [2s; 20s]	7s [6s; 9s]	5s [3s; 8s]			N/A [N/A; N/A]
6	75	59s [8s; 2m]	1m [46s; 2m]	11s [10s; 12s]			N/A [N/A; N/A]
6	100	26s [5s; 3m]	14m [14m; 14m]	5m [5m; 5m]			N/A [N/A; N/A]
8	100	1m [13s; 2h]		3m [1m; 5m]			N/A [N/A; N/A]
10	100			5m [4m; 5m]			N/A [N/A; N/A]

Legend
- Correctly solved
- Incorrectly solved
- Failed
